# Transcriptome-Wide Single Nucleotide Polymorphisms (SNPs) for Abalone (*Haliotis midae*): Validation and Application Using GoldenGate Medium-Throughput Genotyping Assays

**DOI:** 10.3390/ijms140919341

**Published:** 2013-09-23

**Authors:** Aletta Bester-Van Der Merwe, Sonja Blaauw, Jana Du Plessis, Rouvay Roodt-Wilding

**Affiliations:** Molecular Breeding and Biodiversity Group, Department of Genetics, Faculty of Agrisciences, Stellenbosch University, Private Bag X1, Matieland 7602, South Africa; E-Mails: me.sonja.blaauw@gmail.com (S.B.); jdup27@gmail.com (J.P.); roodt@sun.ac.za (R.R.-W.)

**Keywords:** abalone, *Haliotis midae*, SNP validation, transcriptome, GoldenGate assay, medium-throughput genotyping

## Abstract

*Haliotis midae* is one of the most valuable commercial abalone species in the world, but is highly vulnerable, due to exploitation, habitat destruction and predation. In order to preserve wild and cultured stocks, genetic management and improvement of the species has become crucial. Fundamental to this is the availability and employment of molecular markers, such as microsatellites and single nucleotide (SNPs). Transcriptome sequences generated through sequencing-by-synthesis technology were utilized for the *in vitro* and *in silico* identification of 505 putative SNPs from a total of 316 selected contigs. A subset of 234 SNPs were further validated and characterized in wild and cultured abalone using two Illumina GoldenGate genotyping assays. Combined with VeraCode technology, this genotyping platform yielded a 65%–69% conversion rate (percentage polymorphic markers) with a global genotyping success rate of 76%–85% and provided a viable means for validating SNP markers in a non-model species. The utility of 31 of the validated SNPs in population structure analysis was confirmed, while a large number of SNPs (174) were shown to be informative and are, thus, good candidates for linkage map construction. The non-synonymous SNPs (50) located in coding regions of genes that showed similarities with known proteins will also be useful for genetic applications, such as the marker-assisted selection of genes of relevance to abalone aquaculture.

## 1. Introduction

The South African abalone, *Haliotis midae*, is a highly valuable marine resource and one of almost 30 commercially viable abalone species found worldwide [1]. The South African abalone industry, the largest outside Asia, currently has a total output of 1015.44 metric tons, valued at 355 million South African Rand (ZAR) [2]. In order to supply the growing international market and to ensure the sustainability of the industry, effective farm management practices, including the employment of molecular markers in genetic management programs, are vital [3]. To date, mainly microsatellite markers (274) have been developed for *H. midae* using various enrichment and *in silico* techniques [4–7]. Although these markers have already proven to be very useful, they have limitations, including development time, size homoplasy and the presence of null alleles [4]. The focus has therefore shifted towards the development of single nucleotide polymorphisms (SNPs) with 40 SNPs isolated thus far [8,9]. These markers have become increasingly popular in recent years, due to their abundance in genomes, the ease of genotyping and the reduction in development costs [10]. The increase of SNP development in aquaculture species is evident in reports on Atlantic cod (*Gadus morhua*) [11,12], Japanese scallop (*Patinopecten yessoensis*) [13], Atlantic salmon (*Salmo salar*) [14,15], Channel catfish (*Ictalurus punctatus*) [16] and, also, species of abalone, such as Pacific abalone (*Haliotis discus hannai*) [17,18].

In the case of *H. midae*, as in many other non-model organisms, no complete genome map is currently available. Transcriptomic data (in the form of expressed sequence tags (ESTs)), however, serve as a viable source for SNP discovery [8,19–21], facilitating an easier and more cost-effective means of generating genomic resources in non-model organisms [22]. Despite limitations, such as inferring intron positions and genomic gene order, the use of transcribed sequences for marker development proves advantageous, since such markers are directly associated with genes and could be very useful for gene-associated mapping, the identification of causative genes and interspecific transferability between closely related species; an important consideration, especially in non-model species, where knowledge of the genome is limited [23].

Single nucleotide polymorphism isolation procedures have improved greatly, due to advances in DNA sequence technology, and, at present, most commonly include the utilization of whole genome or transcriptome next generation sequencing (NGS) data. The NGS platforms expedite sequence data generation by increasing the production of sequence data to several thousand megabase pairs (Mb) [24,25], and the generated reads allow for efficient assembly of contigs [26,27]. Next generation sequencing has paved the way for sequencing, genotyping and high-throughput marker discovery at an affordable rate [28]. Mining of SNPs from NGS-generated ESTs mainly involves creating, clustering and assembling the generated ESTs, followed by SNP identification by means of *in vitro* or *in silico* approaches [29]. *In vitro* detection of SNPs involves re-sequencing of targeted ESTs to identify nucleotide variations, whereas *in silico* detection refers to the use of bioinformatic pipelines to identify polymorphisms [30,31]. Large-scale data generation methods, such as NGS, coupled with high-throughput genotyping techniques allow for far more efficient means of SNP development and characterization than was previously possible.

Currently, various high-throughput genotyping platforms exist, but only a few are suitable for medium-throughput genotyping, which is preferred for non-model species [32]. In this study, medium-throughput genotyping for SNP characterization was performed using the Illumina GoldenGate genotyping assay with VeraCode technology on the BeadXpress platform. The GoldenGate assay includes locus identification by means of hybridization, enzymatic allele discrimination and exponential amplification of target sequences with the use of three assay oligonucleotides for each SNP [33]. The first two oligonucleotides are allele-specific oligonucleotides (ASO), while the third oligonucleotide, the locus-specific oligonucleotide (LSO), is complementary to the sequence of interest, thus allowing for hybridization downstream from the SNP. Various degrees of multiplexing can be applied to minimize cost and time [34]. The VeraCode technology employs silica glass microbeads inscribed with digital holographic barcodes, which act as solid substrates in solution [35]. All three oligonucleotides are complementary to the universal primers, but the LSO also has a unique address sequence that is complementary to a specific VeraCode bead. The final component is the BeadXpress Reader, a platform with a dual-color laser that scans the microbeads to identify the unique code within each bead.

In this study, we focused on the development of SNPs from transcriptome data previously described for *H. midae* following two bioinformatic pipelines. The EST data formed the basis for *in vitro* and *in silico* SNP discovery. Primer efficiency and genotyping success was evaluated by characterization of two GoldenGate assays in individuals from wild and cultured *H. midae* populations. Downstream applications of successfully genotyped SNPs were also assessed for both GoldenGate assays, and these results form part of an integrative research effort with regards to genetic characterization and improvement of South African abalone. The 48-plex GoldenGate assay (Plex-48) was employed to assess population differentiation between six *H. midae* populations, and a second 192-plex GoldenGate assay (Plex-192) was used to illustrate and test the utility of SNP markers in conjunction with microsatellite markers for linkage map construction in eight *H. midae* full-sib families.

## 2. Results and Discussion

### 2.1. Transcriptome Data and SNP Discovery

The Velvet assembly of transcriptome data utilized in the first bioinformatics analysis yielded 30,689 contigs with a minimum length of 80 bp and an average length of 276 bp. The total number of contigs that resulted from the CLC Genomics Workbench *de novo* assembly was 22,761, with an average length of 260 bp and an average contig coverage of 400 reads/contig. For the *in vitro* SNP detection via re-sequencing of 58 selected contigs, a total of 66% of the optimized PCR amplicons yielded trace quality adequate for putative SNP discovery. The *in vitro* primer success rate (primers that amplified successfully) observed correlated well with similar studies on aquaculture species, including that of the Eastern oyster (*Crassostrea virginica*) (69%) [36], Pearl mussel (*Hyriopsis cumingii*) (58%) [37] and Pacific abalone (*Haliotis discus hannai*) (67.3%) [17]. The general consensus is that stringent parameters and the knowledge of intron-exon boundaries are important considerations for designing primers when utilizing the *in vitro* approach [17,38]. A study in Pacific oyster (*Crassostrea gigas*) also showed a marked (~30%) increase in primer success rate, by designing primers where the reverse primer is situated in the 3′ untranslated region (UTR) [39]. A similar rationale was followed in the current study on the premise that introns are highly infrequent in the 3′-UTR [40]. For the *in silico* detection approach using the SNP detection module in CLC Genomics Workbench, 958 assembled contigs containing 3645 putative SNPs were identified. Following the *in vitro* and *in silico* detection approaches, a subset of 505 putative SNPs (105 *in vitro*, 400 *in silico*) was selected. This amounted to approximately one SNP every 550 bp, which is a much lower SNP frequency than previously obtained in *H. midae* (one SNP every ~150 bp; [8,9]) and in other Haliotid species (1 SNP every ~50 bp; [17,38]). This could possibly be ascribed to the stringent parameters that were set for putative SNP calling, such as the absence of polymorphisms in the 60 bp flanking sequences required for the GoldenGate assays. Studies in catfish found an increase in SNP frequency coupled with an increase of contig size [41], but no association between the fragment length and the number of putative SNPs was observed for this study. A total of 65 (62%) transitions and 38 (36%) transversions were observed for the *in vitro* SNPs, giving an observed transition to transversion (ts:tv) ratio of 1.71, while 253 (63%) transitions and 145 (36%) transversions for the *in silico* SNPs, representing a ts:tv ratio of 1.74 (Table 1). Although the overall ts:tv ratio of 1.74 observed in the current study was slightly higher than previously obtained for *H. midae* [7,11,12], a high ts:tv ratio in general is a good measure for a low frequency of false positives in SNP development and confirmed a high validation rate for the selected SNPs in this study [42].

### 2.2. SNP Performance

Due to the various SNP isolation methods used in this study, it was necessary to evaluate the different approaches in terms of marker performance. The SNPs evaluated in Plex-48 were isolated in four different ways: (1) directly from a cDNA library via a targeted EST approach (8%), (2) from microsatellite flanking regions (17%), (3) from EST contigs and re-sequencing (*in vitro*: 50%) and (4) via the CLC workbench approach (*in silico*: 25%). The loci included in Plex-192 were all detected using the CLC workbench SNP detection utility. It was found that the majority (57%) of SNPs that failed to genotype were identified from microsatellite flanking regions. This failure could be due to the hyper-variability of microsatellite flanking regions and, consequently, the possibility that primers are binding in variable regions [9]. The success of SNPs isolated *in silico* depends mostly on the species, the representative sample used for generating data, as well as the quality of the sequencing data [10,41,43]. In this study, sequencing data with high representation and depth [44] was used in order to identify candidate SNPs with high confidence, and even though 17% of the *in silico* markers from Plex-48 and 23.7% of the markers from Plex-192 failed to genotype, this method proved to be a more time- and cost-effective means of isolating SNPs in a non-model species.

### 2.3. Genotyping Success of GoldenGate Assays

**Figure 1:**
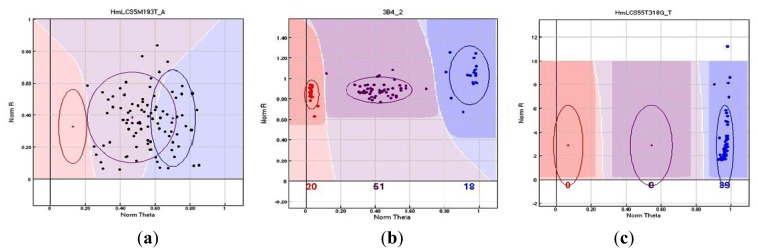
Genoplots obtained with the GenomeStudio™ Genotyping Module representative of (**a**) a failed SNP; (**b**) a successfully genotyped (polymorphic) SNP; and (**c**) a monomorphic SNP analyzed in this study.

An overall (global) genotyping success rate of 85.4% (41 SNPs) and 76.3% (142 SNPs) was obtained for Plex-48 and Plex-192, respectively. Of the successfully genotyped loci, a total of 159 SNPs were found to be polymorphic and 24 were monomorphic (Table 2). These results were based on allele calls obtained with the GenomeStudio™ Genotyping Module in which genotypes are generated for individuals at each locus as genoplots. A GenCall score was subsequently generated to represent the clustering of each individual SNP and, thus, the reliability of each genotyping score [45]. Scores ranged between zero and one and a score closer to one indicated that the genotype inferred was reliable, which could be visually verified with the genoplots (Figure 1). Lower GenCall scores were less reliable and indicated a clear separation from the center of the cluster. Failed SNPs could not be assigned to a genotypic cluster, due to low GenCall and GenTrain (<0.25 for Plex-48; <0.45 for Plex-192) scores depicted in Figure 1a. Sanger sequencing of randomly selected SNPs from Plex-48 confirmed the accuracy and reliability of the calls made by the GenomeStudio module. Polymorphic (Figure 1b) and monomorphic loci (Figure 1c) could also be distinguished through variation in the clustering. Only SNPs consisting of a ≥0.80 GenTrain score and a ≥80% call rate, as well as a minor allele frequency (MAF) of ≥0.01 were considered to be successfully genotyped.

In this study, only SNPs with a functionality score ≥0.75 were selected for validation to ensure a high genotyping success rate, but despite this, 14.6% (7 from Plex-48) and 22.9% (44 from Plex-192) still failed to cluster and were considered genotyping failures. Previous studies found that the inclusion of SNPs with functionality scores ≤0.6 reduced the overall success rate of such markers significantly [13,46]. In Plex-48, the recommended GenTrain cut-off <0.25 [47], indicating a low call rate, was utilized. For Plex-192, a more stringent GenTrain cut-off of <0.45 was used, while a GenCall rate ≥80% was applied in both assays.

The success of any genotyping method is reflected in what is referred to as the conversion rate and the global success rate. The former considers only the polymorphic markers, whereas the global success rate considers all the markers (monomorphic and polymorphic) that were successfully typed within the sample group [13]. In this study, a global success rate of 85.4% for Plex-48 and 76.3% for Plex-192 proved to be relatively high and corresponds to that obtained for other non-model species, such as, for example, catfish (*Ictalurus* spp.—69%; [44]) and maritime pine (*Pinus pinaster*—66.9%; [13]. The conversion rate obtained for Plex-48 (65.4%) and Plex-192 (69.4%) was also in accordance, e.g., with catfish (*Ictalurus* spp.—59.8%; [41]) and the white- (*Picea* glauca—69.2%) and black spruce (*Picea mariana*—77.1%) [46]. The conversion rates for the current study fall short when compared to the manufacturers’ predictions (93%); however, it must be noted that these predictions are based on research in model species [48]. Factors that may have attributed to the lower conversion rates are the extreme GenTrain cut-off values applied in the current study, the presence of paralogous SNPs or limited knowledge regarding the complexity of the abalone genome.

Final confirmation of the success of the current genotyping assays is the significantly lower number of monomorphic SNPs (24% and 7.5%, for Plex-48 and Plex-192, respectively) that was found in comparison to other aquaculture species [41]. Interestingly, some markers were found to be heterozygous in all individuals, which could be due to cross-amplification of the allele-specific primers [36] and could represent paralogous duplications, a phenomenon already discovered in the sperm lysin gene in *H. tuberculata coccinea* [49]. These markers were not utilized in further applications.

### 2.4. Functional Annotation and SNP Effect

Functional annotation of the contigs utilized in the compilation of the GoldenGate assays (Plex-48 and Plex-192) indicated that the majority of the sequences showed significant similarity to the genes of interest: 88% (Plex-48, Table 3) and 79% (Plex-192). Further detail of annotation, sequence similarity (*E*-value), expected variants and SNP position and the effect for SNPs of Plex-48 and Plex-192 are shown in Supplementary Tables S1 and S2, respectively.

The majority of hits were classified in the Mollusca (47%) and Chordata (22%) phyla. The group Mollusca was further divided into Gastropoda and Bivalvia, with twice as many significant hits classed as Gastropoda. A total of 31 contigs could not be annotated, and the position and functional effect for four Plex-48 SNPs (8.33%) and 30 Plex-192 SNPs (16.13%) could, therefore, not be determined. For the two assays combined, 34.61% of the SNPs were located in the 3′ untranslated regions (UTRs), while 50.85% of the SNPs were in coding regions. Further analysis of functional changes revealed that 69 of the total SNPs were synonymous and 50 were non-synonymous, of which the latter accounted for over 20% of the SNPs developed in this study and could be considered as functionally important changes in the corresponding proteins [50].

### 2.5. SNP Diversity, Population Differentiation and Family Informativeness

All SNP loci showed two alleles and were in agreement with those originally observed before validation (Table 4, Table S3). Of the 159 polymorphic SNPs, another 13 were in fact monomorphic in the subsamples selected for estimation of genetic diversity parameters. Among the rest, MAF ranged from 0.0014 to 0.4781 for Plex-48 with a mean of 0.1542 (Table 4), while for Plex-192, MAF ranged from 0.0417 to 0.5 with a mean of 0.2086 (Table S3). Observed (H_o_) and unbiased expected heterozygosity (H_e_) values ranged from 0.003 to 0.788 and 0.003 to 0.497, respectively, for Plex-48 and from 0.083 to 0.750 and 0.083 to 0.522 for Plex-192. For Plex-48, 15 SNP loci deviated significantly from Hardy-Weinberg equilibrium (HWE) (*p* < 0.05), while only 12 SNPs were not in accordance with HWE for Plex-192. The low to moderate levels of heterozygosity along with the average MAF (18.1%) observed in the majority of the SNPs applied in this study were comparable to reports in, for example, Pacific abalone, *H. discus hannai* [17,18] and turbot, *Scophthalmus maximus* [51]. Although the average MAF was indicative of a fairly significant degree of homozygosity, a low inbreeding coefficient (average *f* = −0.188) was observed for all the Plex-48 SNPs and most of the SNPs of Plex-192.

**Figure 2:**
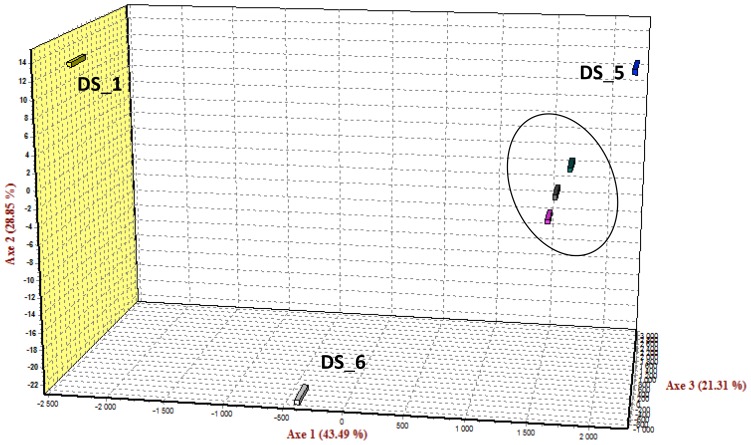
Factorial component analysis (FCA) based on 31 SNP loci in six *H. midae* populations. The respective populations are indicated by color: Family DS_1 (Yellow), Family DS_5 (Blue), Family DS_6 (White), Riet Point (Grey), Saldanha Bay (Pink) and Witsand (Green). The wild populations are encircled.

Applying the 31 polymorphic markers of Plex-48, population differentiation between three wild and three commercial populations was inferred based on summary statistics (Wrights’ *F*-statistics), as well as multivariate analysis (FCA). Pairwise *F*_ST_ (θ) values ranged from 0.006 to 0.199, with significance (*p* < 0.05) over all but one of the population pairs tested. Results indicated limited genetic differentiation between the three wild populations, while the relatively high values (*F*_ST_ = 0.090–0.140) obtained between wild and cultured populations indicated considerable genetic differentiation between wild and cultured abalone. Factorial correspondence analysis was performed to obtain a three-dimensional view of the genetic relationship between the six different populations. The first factor accounted for 43.39% of the genetic variation and the second factor for 24.02%. Most evident from the FCA plots was the tight clustering of the three wild populations and the noticeable genetic distinctness of each of the three cultured populations (Figure 2). Comparison of the individual genotypes supported the substantial overlap of individuals from wild populations, while virtually no overlapping was observed between individuals of the three cultured populations. Population analysis indicated that allele fixation (increasing *F*_ST_) was evident between cultured and wild populations, but was most striking between the three cultured full-sib families. The differentiation between wild and cultured abalone was in accordance with what has recently been found in *H. midae* based on microsatellite markers [3], while the unusually high variation observed between the cultured populations (pairwise *F*_ST_ > 0.15) could be attributed to the highly heterozygous nature of the wild-caught parents, leading to an increase in the probability of the resulting F1 progenies receiving two different alleles at each locus, inflating the genetic distinctness of the respective families. With regards to the wild populations that included abalone from the West (Saldanha Bay), South (Witsand) and East coast (Riet Point) of South Africa, FCA results corroborated the summary statistics in that the only statistically unsupported pairwise *F*_ST_ value was found between the wild populations of Witsand and Saldanha Bay. Bester-van der Merwe *et al.* [52] previously reported that there was low, yet significant, differentiation between wild populations of *H. midae*, but that the primary barrier to gene flow was around the Cape-Agulhas area. Saldanha Bay is situated west of Cape-Agulhas and Witsand and Saldanha Bay, east of the proposed barrier. The level of population differentiation and pattern of gene flow depicted by the SNPs in this study are therefore in full agreement with the population structure previously suggested for wild *H. midae* populations. The population differentiation was also supported by the molecular analysis of variance (AMOVA) results, with significant differentiation amongst populations within groups (*F*_SC_ = 0.141, *p* = 0.000) and within populations (*F*_ST_ = 0.141, *p* = 0.000).

Mendelian segregation analysis showed that only a small percentage of markers did not adhere to Mendelian patterns of inheritance in the respective mapping families, and the highest percentage (20%) was exhibited for family DS_1. Only a very small number of SNPs (19 across all families) showed distortion of segregation after correction for multiple tests, and most of the markers were distorted in one family only. Consequently, a high percentage of the SNPs (118) developed in this study were found to be informative markers (heterozygous in both or at least one of the parents) and could be considered as candidates for linkage map construction [53]. However, due to their bi-allelic nature, a larger number of SNP markers *versus* microsatellites should be included in a linkage mapping study, and these markers should initially be genotyped in the parents to ensure that less of these markers will need to be excluded. This would ensure the inclusion of only informative markers in linkage map construction without the need to discard monomorphic markers. In addition, testing for non-Mendelian segregation of the SNPs is also useful in detecting the presence of null alleles [50]. According to the Mendelian ratios obtained in this study, possible null alleles were present for only a small percentage of markers, confirming their usefulness in future applications.

## 3. Experimental Section

### 3.1. Transcriptome Sequencing and SNP discovery

Samples and methods for sequencing and *de novo* assembly of the *H. midae* transcriptome have been described in detail by Franchini *et al.* [44]. In brief, this included RNA extraction from 19 animals from a single family, followed by cDNA library construction and sequence generation by the Illumina Genome Analyzer (GA II). For EST characterization and identification of SNPs, contig assembly and annotation were performed utilizing two separate bioinformatic analyses. The first analysis involved the use of Velvet 0.7.52 for contig assembly and the cDNA Annotation system (dCAS) 1.4.3 for annotation of contigs. In the second analysis, high quality reads were assembled *de novo* using the CLC Genomics Workbench v4.0 software (CLCbio, Aarhus, Denmark), and sequence annotation was performed using Blast2GO 2.4.4 [54]. In both analyses, the databases against which the annotation was completed included the eukaryote clusters of genes (KOG; [55]), Gene Ontology (GO; [56]) and the Kyoto Encyclopedia of Genes and Genomes (KEGG; [57–59]).

For the *in vitro* identification of SNPs, annotated contigs resulting from the first bioinformatic analysis were screened manually to identify contigs with significant hits (*E*-value < 1.0 × 10^−17^) against genes with known functions using the BLASTN algorithm (BLAST) [60]. A set of 58 annotated contigs (*E*-value < 1.3 × 10^−19^) were selected and facilitated the design of 97 primer pairs with BatchPrimer3 [61]. Twenty of the 58 contigs were fragmented into smaller sections of approximately 700 bp to ensure amplification of the entire contig with internal primers. Successful primers were used to amplify the genomic DNA of eight unrelated *H. midae* individuals in 10 μL reaction volumes containing 20 ng genomic DNA, 200 μM dNTPs, 2.0 mM MgCl_2_, 2.0 pmol of each primer and 0.25 U GoTaq^®^ Flexi DNA polymerase (Promega, Madison, WI, USA). Thermal cycling was conducted on the Gene-Amp System 2700 thermal cycler (Applied Biosystems, Foster City, CA, USA), and conditions consisted of an initial denaturing step of 95 °C for 5 min, followed by 35 cycles of 94 °C for 30 s, Tm (specific for each primer pair) for 45 s, 72 °C for 45 s and a final elongation step of 10 min at 72 °C. Assessment of PCR amplification was conducted through 2% agarose gel electrophoresis. PCR amplicons were purified, quantified and Sanger sequenced using the BigDye^®^ Terminator v3 Cycle Sequencing kit (Applied Biosystems) and the ABI PRISM^®^ 3100 DNA automated sequencer (Applied Biosystems). DNA sequence chromatograms were individually analyzed using Sequence Scanner v1.0 (Applied Biosystems) to determine sequence quality. Multiple alignments for sequences of each primer pair were carried out using ClustalW v1.4 [62], implemented in BioEdit v7.0.0 [63]. Sequence similarity was determined with BLASTN to assess if the correct amplicons were amplified. Alignments and chromatograms of all eight individuals were visually screened for putative SNPs. Only base positions with two peaks, of which the height ratio was approximately ≥1:2, were considered as putative SNP loci for heterozygous individuals.

For the *in silico* identification of SNPs, the SNP detection module in CLC Genomics Workbench was used to screen 22,761 assembled contigs following the second bioinformatic pipeline. The parameters that were set for putative SNP calling included a minimum quality score of 20, minimum coverage of 80 and a minor allele frequency (MAF) >10%. Of these, 139 SNP-containing contigs were selected for further analysis of 400 putative SNPs. To ensure reliable primer design, the flanking sequences of putative SNPs were checked to ensure that there were no other polymorphisms within 60 bp of the SNPs. Contigs containing the predicted SNPs were then subjected to BLAST similarity searches using Blast2GO.

### 3.2. SNPs and Samples for GoldenGate Assays

SNP characterization using the GoldenGate genotyping assay was conducted in two separate multiplex assays, further referred to as Plex-48 and Plex-192. The SNPs selected for Plex-48 were comprised of 24 *in vitro* SNPs developed in the first bioinformatic analysis, 12 *in vitro* SNPs previously developed by Bester *et al.* (2008) and Rhode (2010) and 12 novel *in silico* SNPs identified by means of the second bioinformatic pipeline using CLC Genomics Workbench. The *in silico* SNPs represented a test panel to determine the efficiency and performance of markers identified with the CLC workbench. For Plex-192, 186 SNPs from the second bioinformatic pipeline and six SNP loci from Plex-48 (positive SNP controls) were included. For both assays, sequences containing putative SNPs were submitted to the Illumina Assay Design Tool to determine the functionality and primer designability score for each SNP locus. Scores of 0.5–1.0 are required for a high quality assay, and only loci with a final score ≥0.75 were considered for genotyping.

Samples for Plex-48 included individuals from three wild abalone populations from the coast of South Africa (Saldanha Bay, Witsand and Riet Point), as well as parents and offspring of four linkage mapping families collected from two commercial abalone farms (Table 5). The samples used for Plex-192 included individuals from six linkage mapping families reared on two commercial farms, two of which overlapped with the cultured populations used in Plex-48 (Table 5). DNA samples were precipitated and resuspended in TE Buffer (1 M Tris-HCl, 0.5 M EDTA, water at pH 8.0) or DNase-free water (Promega, Madison, WI, USA) to a final concentration of 50 ng/μL. Each plate from Plex-48 contained two positive and one negative control to monitor genotyping efficiency and possible contamination, and plates from Plex-192 each contained 3 genotyping (positive) controls.

### 3.3. SNP Genotyping and Functional Effect

Genotyping was achieved with the Illumina GoldenGate genotyping assay according to the manufacturer’s specifications. Genotyping was conducted at the National Health Laboratory Services (NHLS) facility at the University of the Witwatersrand, South Africa. The GenomeStudio™ Genotyping Module v1.0 was employed for data analysis to infer genotypes and allele frequencies for each locus. Data quality assessment was done with the default No-Call (GenCall) parameters defined by Illumina, a GenTrain (clustering algorithm) value of 0.25 for Plex-48 and 0.45 for Plex-192 and an individual call rate of 0.85 for Plex-48 and 0.80 for Plex-192. The more stringent GenTrain value used for Plex-192 was due to a lack of clustering taking place at values below 0.45, which led to the exclusion of those markers. Individuals that failed genotyping and SNPs that illustrated ambiguous clustering were omitted prior to further analysis. As an additional means of validation, nine randomly selected SNPs were sequenced in 30 individuals using the ABI PRISM^®^ BigDye Terminator v3.1 Cycle Sequencing kit in the forward direction. Genotypes acquired from the Sanger sequencing were manually compared to those generated by the Genotyping Module. For one of the cultured populations, DS_2, the majority (87.6%) of the population could not be genotyped, due to technical difficulties, and were excluded from further analysis.

All the ESTs and contigs screened for SNPs in this study were subjected to BLASTX similarity searches against the NCBI protein database using Blast2GO. Functionally annotated contigs with the highest sequence similarity were used to identify the strand and reading frame orientation. The contig sequences containing SNPs within coding regions, or, alternatively, the corresponding 120 bp flanking sequences, were translated with the EMBOSS transeq utility for all six possible reading frames. The resulting amino acid sequences for alternative alleles at each SNP locus were compared to determine whether SNPs were synonymous or non-synonymous (Tables S1 and S2).

### 3.4. Genetic Diversity, Population and Family Analysis

All successfully genotyped SNPs were further evaluated for the level of polymorphism observed in a subset of individuals that represented wild abalone samples. For Plex-48, this included all the wild populations and the parents of the four full-sib families, while for Plex-192, only the 12 parents (six males and six females) of the six mapping families were included, as they were the only individuals of Plex-192 that could be considered wild abalone. Genetic diversity estimates, such as minor allele frequency, observed and expected heterozygosity and locus-specific *F*_is_, were obtained using GENEPOP 4.0 [64]. Hardy-Weinberg equilibrium (HWE) was also computed via the exact probability test (10,000 dememorizations, 500 batches and 5000 iterations per batch) in GENEPOP.

In order to test for population differentiation between wild and cultured populations (including offspring), the polymorphic markers from Plex-48 were employed to determine the pairwise *F*_ST_ estimator (θ) of Weir and Cockerham [65] in GENETIX 4.04 [66]. Significance was tested with 1000 permutations, and the Bonferroni correction model was used to adjust the significance levels for multiple tests. Population allele frequency data were subjected to factorial component analysis (FCA), also available in GENETIX. This provided a three-dimensional view of the distribution of genetic variation between the six genotyped populations. Molecular analysis of variance (AMOVA, 10,000 permutations) was performed in ARLEQUIN 3.5.1 to further test the grouping hypothesis of wild *versus* cultured populations.

Selected progenies of all six mapping families (two of which were analyzed with both Plex-48 and Plex-192) included in this study were used to evaluate conformance to Mendelian segregation for all the polymorphic SNPs (234). Offspring ranged from 70 individuals for Family H to 103 for Family DS_1. SNP markers were tested for segregation distortion from expected Mendelian ratios with the chi square goodness-of-fit test (*p* < 0.05) and subjected to linkage analysis using JOINMAP^®^ 4.1 [67] to create sex-average and sex-specific maps. Linkage analysis and results are reported in detail by Vervalle *et al.* [53].

## 4. Conclusions

The major objective of this study was to investigate the utility of ESTs generated by Illumina sequencing-by-synthesis for the development of SNPs in the abalone, *Haliotis midae*, since transcriptome sequencing in non-model species promises greater representation of the functional characteristics of these species [30,68,69]. The increased popularity of type I markers has redirected research toward the development of SNPs instead of microsatellites. These SNPs can ultimately be applied in conjunction with microsatellite markers in various applications, such as genetic diversity studies, population structure analyses, linkage mapping, quantitative trait locus (QTL) analysis and parentage assignment. A further objective of this study was therefore to test the utility of the successfully genotyped SNPs in determining genetic diversity, population differentiation and family informativeness in *H. midae*, since previously, these types of applications were addressed mainly using microsatellite markers [3,5,55,70]. In this study, transcriptome characterization with the aid of NGS technologies proved to be adequate for the use of marker development in *H. midae*. The Illumina GoldenGate assay was equally successful in testing the utility of these markers in population differentiation inference, as well as in the saturation of a preliminary linkage map for *H. midae*, both of which are very important in the genetic management of this South African mollusk. In the current study, contigs for SNP development were selected based on a vast selection of genes of relevance, ranging from cellular processes to stress response, which is potentially also linked to important traits in aquaculture species, such as disease resistance and growth. Further analysis of these markers, specifically focusing on the functional SNPs identified in this study, can be directed to target genes of interest, such as those regulating immune response and environmental adaptation, as well as gene expression studies to facilitate a better understanding of this species’ genome.

## Figures and Tables

**Table 1:** Summary of the variants of the putative single nucleotide polymorphisms (SNPs) detected *in vitro* and *in silico* for *H. midae* following two bioinformatic pipelines.

	*In vitro* (Velvet assembly)	*In silico* (CLC assembly)
**Number of contigs**	58	256
**Number of putative SNPs**	105	400
**Transversions:**		
A/T	15 (14.3%)	52 (13.0%)
A/C	7 (6.7%)	35 (8.8%)
C/G	10 (9.5%)	15 (3.8%)
T/G	6 (5.7%)	43 (10.8%)
**Transitions:**		
A/G	35 (33.3%)	124 (31.0%)
T/C	30 (28.6%)	129 (32.3%)
**Other**	2 (1.9%)	2 (0.5%)

**Table 2:** Comparison of the genotyping success of two GoldenGate assays (Plex-48 and Plex-192) assembled for *H. midae*. EST, expressed sequence tag.

	Plex-48	Plex-192
Functionality score	0.75	0.8
ESTs/Contigs	35	139
Feasible SNPs	48	186
GenTrain score	0.25	0.45
Total	48	186
Failures	7 (14.58%)	44 (23.7%)
Monomorphic	10 (20.83%)	14 (7.5%)
Polymorphic	31 (64.58%)	128 (68.8%)

**Table 3:** Annotation, variants and predicted gene location and functional effect of the 48 feasible SNPs validated in the GoldenGate genotyping assay, Plex-48. UTR, untranslated region.

SNP Name	EST/Contig	Functional annotation	Variant	SNP effect
*3B4_2*	3B4	60s Ribosomal protein L8	T/C	UTR
*3B4_7*			A/T	UTR

*3D10_1*	3D10	Hemocyanin	A/G	UTR

*2H9_2*	2H9	Ribosomal protein L22	A/T	UTR

*HdSNPc148_820T_C*	HdSNPc148	Actin	T/C	Synonymous

*HdSNPc106_688C_T*	HdSNPc106	Tubulin alpha-1a chain isoform 2	C/T	Synonymous

*HmSNPc4_815C_T*	HmSNPc4	Microsatellite sequence	C/T	Non-synonymous

*HaSNPdw500_207C_T*	HaSNPdw	Microsatellite sequence	C/T	UTR

*HmLCS5M193T_A*	HmLCS5M	-	T/A	-

*HmLCS5M479C_T*		Opacity protein	C/T	Synonymous

*HmLCS55T318G_T*	HmLCS55T	Microsatellite sequence	G/T	Non-synonymous

*HmRS36T262T_C*	HmRS36T	5-Formyltetrahydrofolate cyclo-ligase	T/C	Synonymous

*SNP101_113*	Contig 101	Myosin heavy chain	A/C	UTR
*SNP101_201*			C/G	UTR

*SNP146.2_132*	Contig 146	ADP/ATP carrier protein	A/G	Synonymous
*SNP146.3_123*			T/G	Non-synonymous

*SNP149.1_106*	Contig 149	Heat shock protein 70	A/C	UTR
*SNP149.1_374*			C/G	Non-synonymous
*SNP149.2_165*			A/G	Synonymous
*SNP149.4_75*			A/G	UTR
*SNP149.4_341*			T/C	UTR

*SNP210_266*	Contig 210	-	T/G	-

*SNP214_86*	Contig 214	Ribosomal protein L10	T/C	Non-synonymous
*SNP214_434*			T/C	UTR

*SNP342.2_537*	Contig 342	Heat shock protein	T/C	UTR

*SNP449.2_110*	Contig 449	s-Adenosylmethionine synthetase isoform type-1	A/G	Non-synonymous
*SNP449.2_443*			T/C	Synonymous

*SNP1718_109*	Contig 1718	NADH dehydrogenase subunit 1	A/T	UTR

*SNP1833_160*	Contig 1833	Alpha tubulin	A/G	UTR

*SNP1834_76*	Contig 1834	Tubulin alpha-1a chain- partial	A/G	UTR
*SNP1834_464*			A/G	Non-synonymous

*SNP1949_235*	Contig 1949	Ribosomal protein L3	A/C	UTR

*SNP4691_183*	Contig 4691	Heat shock protein 70	A/G	UTR

*SNP17550.1_463*	Contig 17550	Clathrin heavy chain 1	A/G	Non-synonymous
*SNP17550.3_221*			A/T	UTR
*SNP17550.3_555*			A/T	UTR

*SNP48_322*	Contig 48	Collagen alpha-4 chain	T/G	UTR

*SNP67_164*	Contig 67	Collagen alpha-6 partial	A/G	UTR

*SNP140_2421*	Contig 140	Na^+^ K^+^-ATPase alpha subunit	T/C	Synonymous

*SNP229_2772*	Contig 229	14-3-3 Zeta	T/C	UTR

*SNP300_1828*	Contig 300	-	A/G	-

*SNP972_1055*	Contig 972	Myosin heavy chain	T/C	Synonymous

*SNP1001_388*	Contig 1001	Cre-sca-1 protein	T/C	UTR

*SNP2091_264*	Contig 2091	-	A/C	-

*SNP3129_923*	Contig 3129	Arginine kinase	A/G	Synonymous

*SNP5837_204*	Contig 5837	Mucus-associated protein partial	T/C	Non-synonymous

*SNP13865_165*	Contig 13865	Cathepsin l	T/C	UTR

*SNP20648_3041*	Contig 20648	Filamin-c isoform 4	A/G	Synonymous

**Table 4:** Genetic diversity estimates of the polymorphic SNP markers (Plex-48) for *H. midae* individuals. HW, Hardy-Weinberg.

SNP Name	Minor allele frequency	Heterozygosity		Inbreeding coefficient	Probability HW
		Observed	Expected		
*3B4_2*	0.4549	0.592	0.465	−0.274	0.01
*3B4_7*	0.1901	0.537	0.473	−0.134	<0.01
*3D10_1*	0.0391	0.281	0.472	0.406	0.001
*HdSNPc148_820T_C*	0.4564	0.183	0.172	−0.066	0.000
*HdSNPc106_688C_T*	0.0015	0.721	0.462	−0.563	<0.01
*HmRS36T262T_C*	0.0420	0.009	0.331	0.972	0.002
*SNP101_113*	0.0557	0.069	0.073	0.049	0.530
*SNP101_201*	0.0015	0.091	0.126	0.278	0.786
*SNP146.2_132*	0.1352	0.285	0.245	−0.165	0.011
*SNP149.1_374*	0.0651	0.025	0.031	0.189	0.799
*SNP149.2_165*	0.4079	0.483	0.478	−0.009	0.001
*SNP149.4_75*	0.2464	0.003	0.003	0.000	-
*SNP210_266*	0.0669	0.044	0.043	−0.021	1.000
*SNP214_86*	0.0000	0.041	0.040	−0.019	0.875
*SNP342.2_537*	0.1023	0.098	0.111	0.116	0.793
*SNP449.2_110*	0.4035	0.32	0.497	0.357	0.000
*SNP1834_76*	0.4781	0.013	0.012	−0.005	-
*SNP1834_464*	0.0014	0.788	0.489	−0.614	<0.01
*SNP1949_235*	0.1764	0.309	0.266	−0.162	0.000
*SNP4691_183*	0.0667	0.138	0.353	0.610	0.025
*SNP17550.3_221*	0.0189	0.013	0.013	−0.005	-
*SNP17550.3_555*	0.0841	0.119	0.112	−0.062	0.081
*SNP67_164*	0.0696	0.182	0.176	−0.033	0.181
*SNP140_2421*	0.1272	0.178	0.162	−0.096	0.310
*SNP229_2772*	0.0338	0.099	0.128	0.228	0.880
*SNP300_1828*	0.0678	0.195	0.181	−0.075	0.058
*SNP972_1055*	0.2267	0.082	0.394	0.794	<0.01
*SNP1001_388*	0.1893	0.280	0.250	−0.119	0.000
*SNP2091_264*	0.2580	0.314	0.486	0.354	0.001
*SNP3129_923*	0.2246	0.318	0.462	0.312	<0.01
*SNP20648_3041*	0.0889	0.164	0.166	0.015	0.063

-no *p*-value was assigned.

**Table 5:** Sample sizes of *H. midae* populations genotyped in Plex-48 and Plex-192.

Sample origin	Number of individuals	
	Plex-48	Plex-192
Family DS 1	103	70
Family DS_2	94	87
Family DS_5	90	-
Family DS_6	94	-
Family D	-	72
Family H	-	71
Family I	-	81
Family J	-	72
Saldanha Bay	23	-
Witsand	26	-
Riet Point	26	-
Positive controls	2 per plate	3 per plate
Negative controls	-	1 per plate

## Supplementary Files

Supplementary File 1 Supplementary Table S3 (XLSX, 39 KB)
